# Dissemination Pattern of Hospital-Acquired Methicillin-Resistant *Staphylococcus aureus* and Community-Acquired MRSA Isolates from Malaysian Hospitals: A Review from a Molecular Perspective

**DOI:** 10.21315/mjms2023.30.2.3

**Published:** 2023-04-18

**Authors:** Nor Syaza Syahirah Amat Junaidi, Nik Noorul Shakira Mohamed Shakrin, Mohd Nasir Mohd Desa, Wan Md Zin Wan Yunus

**Affiliations:** 1 Faculty of Medicine and Defence Health, Universiti Pertahanan Nasional Malaysia, Kuala Lumpur, Malaysia; 2 Centre for Tropicalization, Universiti Pertahanan Nasional Malaysia, Kuala Lumpur, Malaysia; 3 Department of Biomedical Science, Faculty of Medicine and Health Sciences, Universiti Putra Malaysia, Selangor, Malaysia; 4 Laboratory of Halal Science Research, Halal Products Research Institute, Universiti Putra Malaysia, Selangor, Malaysia

**Keywords:** methicillin-resistant Staphylococcus aureus, MRSA, molecular evolution, molecular epidemiology, SCCmec typing, spa typing

## Abstract

The global emergence of methicillin-resistant *Staphylococcus aureus* (MRSA) that unsusceptible to a wide selection of antimicrobial agents and any newly introduced antimicrobial over the past decades has triggered more extensive holistic measures to put an end to this situation. Molecular surveillance of MRSA clones is important to understand their evolutionary dynamics for investigating outbreaks, propagating precautionary measures, as well as planning for appropriate treatment. This review includes peer-reviewed reports on the molecular characterisation of clinical *Staphylococcus aureus* isolates within Malaysian hospitals from year 2008 to 2020. This work highlights the molecular clones of hospital-acquired MRSA (HA-MRSA) and community-acquired MRSA (CA-MRSA) isolates from Malaysian hospitals, with description on their ever-changing pattern. Among HA-MRSA, the ST22-t032-SCC*mec* IV MRSA clone was reported to supplant the previous dominating clone, ST239-t037-SCC*mec* III. Meanwhile, ST30, ST772, ST6 and ST22 were repeatedly detected in CA-MRSA, however, none of the strains became predominant. Future in-depth study on molecular epidemiology of MRSA clone is essential for the investigation of the extent of the clonal shift, especially in Malaysia.

## Introduction

*Staphylococcus aureus* is a Gram-positive bacterium known both as normal flora and a human pathogen that causes mild to life-threatening infections, such as skin and soft tissue infection (SSTI), food poisoning, pneumonia, infective endocarditis, osteomyelitis and bacteraemia (1). This bacterium possesses several exoproteins and toxins to adapt to a host and induce diseases. One of the important toxins is Panton-Valentine leukocidin (PVL). The PVL is a toxin composed of bicomponents, LukS-PV and LukF-PV, encoded by *lukS-PV* and *lukF-PV* genes, respectively. Both of these two components are secreted before they are assembled into a pore-forming heptamer on neutrophil membranes, leading to neutrophil lysis. Normally, this toxin is linked to skin and soft tissue infection, community-acquired infection, recurrent infection without predisposing factors, and severe infections such as necrotising pneumonia and severe musculoskeletal infection (2, 3). Other virulence factors include staphylococcal enterotoxin A to E (encoded by *sea*, *seb*, *sec*, *sed* and *see* genes), protein A (*spa* gene), exfoliative toxin (*eta* and *etb* genes), alpha-haemolysin and delta-haemolysin (*hla* and *hld* genes) and toxic shock syndrome toxin 1 (*tst* gene) (4). The virulence factors contribute to the pathogenicity of staphylococcal foodborne disease and toxic shock syndrome, pathogen binding to Fc domain of host immunoglobulin G, association with staphylococcal scalded skin syndrome (SSSS), cytolytic toxin particularly to human platelets and monocytes, cytolytic activity with high affinity to leucocytes, thereby causing toxic shock syndrome (TSS) and SSTI (4).

Apart from the abovementioned virulent determinant genes, *Staphylococcus aureus* genetic components also comprise antibiotic resistance genes that are mostly encoded on mobile genetic elements (MGEs) (5). Normally, they are defined as DNA fragments that encode a range of virulence and resistance factors, along with enzymes that initiate their transition and incorporation into new host DNA. The MGEs in *Staphylococcus aureus* consist of staphylococcal cassette chromosome (SCC), transposons, integrative conjugative elements (ICEs), integrons, bacteriophages, pathogenicity islands and plasmids (4, 6). These MGEs are movable among bacteria through horizontal gene transfer (HGT) and are believed to be the main reason behind *Staphylococcus aureus* environmental adaptation (4, 6). Examples of such *Staphylococcus aureus* adaptability are the acquisition of resistance to newly introduced drugs and the continuous emergence of new strains.

One key feature that renders *Staphylococcus aureus* infection a major concern is its ability to acquire resistance to the newly introduced drug. Formerly, penicillin was the drug of choice to treat *Staphylococcus aureus* infections. Then, this pathogen developed resistance to penicillin after a year of its usage. Methicillin-resistant *Staphylococcus aureus* (MRSA) emerged after a year of the introduction of semisynthetic antibiotic methicillin. MRSA is referred to the reduced susceptibility of *Staphylococcus aureus* to methicillin and other beta-lactam-related drugs by acquiring SCC *mec* (SCC*mec*) elements in the bacterial chromosome (7). The SCC*mec* includes the *mecA* gene and cassette chromosome recombinases (*ccr*) gene that encodes methicillin resistance property and recombinases for site-specific integration into the bacterial chromosome. The *mecA* gene encodes for a variant of penicillin-binding protein 2A (PBP2A) that may resist beta-lactam inhibition of cell wall synthesis (7). MRSA first emerged in the 1960s in Asian countries (8), whereas MRSA isolates were first reported in 1971 in Malaysia at a university hospital (9). Based on the mode of acquisition, MRSA can be classified as hospital-acquired MRSA (HA-MRSA) and community-acquired MRSA (CA-MRSA).

Another example of *Staphylococcus aureus* adaptability is the blurred line between the distinctive genetic characteristics of HA-MRSA and CA-MRSA. The ST22-SCC*mec* IV strain was originally considered a CA-MRSA strain at its early emergence and has long become an epidemic in hospital settings worldwide. In the early days of its discovery, CA-MRSA was usually associated with the PVL gene rather than HA-MRSA (10). Due to the distinctive genetic characteristics of the MRSA isolates based on the PVL status and the evidence of leucocidin causing increased virulence in the *Staphylococcus aureus* strain, the PVL gene was typically incorporated in the molecular typing of MRSA isolates (11). However, the PVL gene has lost its exclusivity to CA-MRSA upon reports of the latter harbouring the gene (12). This dynamic clonal change of MRSA strains could also change the pathogenesis of the infections and their response to treatments. Hence, it is critical to study the dynamic changing epidemiology of MRSA to evaluate the effectiveness of the existing control measures, gain insight into its evolutionary mechanism and limit its spread.

Further to the above, several MRSA surveillance networks have been established worldwide to control the dissemination of MRSA based on the antibiotic resistance profile and molecular background (13–18). The World Health Organization (WHO) initiated the Global Antimicrobial Resistance Surveillance System (GLASS) in 2014 (19) with the aim to harmonise the established surveillance systems, particularly on antibiotic resistance organisms, and undoubtedly, the antibiotic resistance data from Malaysian hospitals as well. Similarly, Malaysian MRSA antibiotic resistance prevalence is accumulated and reported nationally via the Malaysian National Surveillance on Antimicrobial Resistance (NSAR) (20). In Malaysia, molecular-based surveillance is maintained at small-scale initiatives by several hospitals and laboratories (21–26).

There are several documented events on the rise, decline and replacement of predominant MRSA strains. For example, in Hungary, ST239-SCC*mec* III was the most predominant clone during the 1990s. In the early 2000s, the clone disappeared and was replaced by ST228-SCC*mec* I and epidemic ST5-SCC*mec* II clones (27). The distribution of MRSA clones in Malaysia has been well explored; however, summary of MRSA clonal changes has never been collectively examined. As such, this review describes the distribution and possible clonal changing pattern of Malaysian hospital on MRSA isolates through a summary of published literature on MRSA clones. This review includes studies that reported the molecular characterisation of clinical *Staphylococcus aureus* isolates within Malaysian hospitals. Several studies that were published from 2008 until 2020 were searched using the Medline (via PubMed) and Embase databases based on the following terms with the aid of appropriate Boolean operators: methicillin-resistant *Staphylococcus aureus*, MRSA, molecular, clonal, hospital, clinical and Malaysia. The relevance of the abstract or title of the papers obtained to the review topic was evaluated. Additional publications were culled from the bibliographies. Publication reporting molecular typing of MRSA based on SCC*mec* typing, multilocus sequence typing (MLST) and *spa* typing were chosen. For this review, a total of 18 relevant articles were identified and listed in Table 1.

### Clonal Pattern of Hospital Acquired MRSA Clones in Malaysian Hospitals

A total of five (types I, II, III, IV and V) out of 13 SCC*mec* types of MRSA were reported in Malaysia (Table 1). Type III is the most predominant SCC*mec* type among Malaysian clinical MRSA isolates (22, 26, 28–33). Interestingly, SCC*mec* type IV dominated the current Malaysian hospitals MRSA clones.

Initially, only SCC*mec* types III and IV were noted from the earliest MRSA isolates studied in Malaysia (26). From 12 SCC*mec* type IV isolates, two isolates were PVL positive and multidrug sensitive, i.e. typical characteristics of CA-MRSA, indicating the possible establishment of CA-MRSA strain into the clinical strain. Later in 2008, Lim et al. (22) discovered CA-MRSA SCC*mec* type V from isolates at the same hospital. Meanwhile, in the year 2009, type II was reported in Malaysia (31). The study reported that one of the type II isolates was associated with ST239. The same study also claimed that no ST239-II clone was reported in previous studies, including in Japan and Korea where SCC*mec* type II is common. However, a study involving several Korean hospitals MRSA isolates in 2003 and 2004 reported circulation of this clone (34). This clone has been associated with numerous hospital transmissions (35), but this case has yet to be investigated in Malaysia. This could be due to the insignificant number of type II found in the country (10, 21). Interestingly, type II, typical of HA-MRSA strain, was reported to be isolated among CA-MRSA in a study in West Malaysia (36), demonstrating the establishment of HA-MRSA strain in the community setting.

Most studies involving Malaysian clinical isolates inferred that ST239-SCC*mec* type III is the most circulating clone in the hospital setting (10, 24, 29, 30, 33, 37, 38). ST239-III is a single-locus variant of ST8 found to be the predominant clone in most Asian countries, including from neighbouring countries such as Indonesia, Lao People’s Democratic Republic, Vietnam and Thailand (39–43). ST239-III, also known as the Brazilian/Hungarian epidemic clone, was first detected in Brazil in 1995 and later identified in Hungary in 1997, as the name perceived. Between 1998 and 2003, the ST239 strain disappeared from the Hungarian hospitals and was substantially succeeded by ST228-SCC*mec* type I and ST5-SCC*mec* type II (27).

Most ST239-III isolates in Malaysia are *spa*-type t037 (25, 29). Interestingly, a study by Lim et al. (33) showed that the *spa*-types among ST239 isolates illustrated diversification with an increase in the year of sampling. While the diversity trend has not been statistically confirmed and emphasised in the study, continuous clone surveillance should be performed to elucidate the diversification of MRSA clones in the country. Since 2007, the variability of ST239 in Singapore was again observed following the occurrence of ST22 supplanting ST239 in the early 2000s (44). Moreover, increased diversity of MRSA clones was observed in countries with low MRSA incidence (45). The ease of travelling may aid in the dissemination of the clone into multiple geographical areas, besides the intense selective pressure of the clone itself through mutation in the capsule gene, acquisition of the resistance gene, acquisition of the toxin gene, acquisition of the mobile genetic element and host population factors (46, 47).

Apart from ST239, ST22-IV was also described among the isolates since 2003. Subsequently, detection of ST22 remained in low incidence except in two different studies in 2008 and 2010 (25, 31) where no ST22-IV was isolated. ST22-IV, or epidemic MRSA-15 (EMRSA-15), is a dominant clone regularly found in the UK. This clone can spread and establish itself as the most circulating clone, where it displaced the Iberian and Brazilian clones, the clones commonly found among MRSA isolates in Europe. The clone is particularly interesting because it was also the case in the neighbouring country, Singapore, where ST22 superseded ST239 as the standard sequence type in the country. This phenomenon also occurred among the Malaysian clinical isolates. In a study involving MRSA clinical isolates in 2014 and 2015, ST22-IV was the most prevalent clone identified instead of ST239-III (23). However, a more recent study found that SCC*mec* IV and t032 predominated the MRSA isolates in 2017 (24). Based on the *spa*-typing database (https://www.spaserver.ridom.de/), the MLST of the t032 is found to be ST22. Hence, this further supports the replacement of MRSA clones. The smaller SCC*mec* type IV elements and the specific virulence factors harboured by this clone promote pathogenicity and persistence. However, such a molecular trend was reported in a single tertiary hospital. Hence, there is a need for continuous molecular surveillance of MRSA with a wider geographical coverage to establish the possible dissemination of the clone in Malaysia.

Additionally, there were other common HA-MRSA clones reported among HA-MRSA infections, such as ST45-IV (Berlin clone), ST5-t002 (paediatric clone), ST30-IV (Southwest Pacific clone) and USA-400 (ST1-V), originally assigned as CA-MRSA clones. In Vietnam, ST5 and ST45 were reported among the CA-MRSA isolates (48). Fortunately, these clones have never become pandemic in the local settings. Table 1 lists the molecular genotypes of Malaysian HA-MRSA.

### Clonal Pattern of Community-Acquired MRSA Clones in Malaysian Hospitals

Based on previous studies, the number of clinical CA-MRSA cases reported in Malaysia varies from 5.6% to 44.4% of the total MRSA clinical isolates from the Malaysian hospitals (8, 12, 15, 32, 49). The SCC*mec* type IV preponderated Malaysian clinical CA-MRSA isolates, except in the studies by Sit et al. (10) and Neela et al. (29) where they reported type V and type III as the most common strains of CA-MRSA clinical isolates.

ST30-SCC*mec* type IV, also called the South-West Pacific clone, is the most predominant clonal type of clinical CA-MRSA. A study by Amit et al. (36) reported *spa*-type t019, which is associated with ST30, in their CA-MRSA infection isolates. ST30-IV-*spa* t019 is a pandemic clone and has been documented to replace the predominant CA-MRSA clones in Argentina, with more aggressive behaviour and higher invasion capacity (50). Fortunately, the distribution of ST30 in Malaysia was sporadic and not persistently detected. No ST30 was isolated from the latest CA-MRSA clinical isolates (23).

Over the past 15 years, ST772-SCC*mec* type V has been persistently observed albeit at low rates in several local clinical studies involving CA-MRSA (Table 1). This multidrug-resistant clone first emerged in Bangladesh and India with the name of Bengal Bay clone, which then disseminated globally, triggering a minor outbreak. Intercontinental clone transmission is typically associated with travel or family contact in Bangladesh and India (47, 51). Moreover, sporadic transmission of ST772-V has been reported in several countries, such as Taiwan, Japan, China, Singapore and Myanmar (51, 52) but its persistence among Malaysian isolates should have raised the alarm, as this clone was previously reported causing hospital and household outbreaks in Norway, where MRSA incidence is low (51). A similar situation occurs in Cambodia, where ST834 and ST121 are considered persistent clones among their children CA-MRSA isolates (53). The two STs were first reported in 2009 and consistently detected in the same population in 2011. In 2011, the ST834 strain was also associated with nosocomial infection, indicating that the initially classified as CA-MRSA strain can adapt in hospital settings.

Other clones associated with community characteristics in the hospital infections are ST6-IV, ST1-V, ST80-IV, ST5-V, ST508, ST239-III and ST22-IV (Table 1). In contrast to HA-MRSA-related epidemic transmission, CA-MRSA-related transmission is usually sporadic and rarely causes epidemic outside the region of origins, such as ST8 or USA300 and ST80 in the USA and Europe (51). This is also true in Malaysia, where most of the clones are detected sporadically in low counts. The appearance, disappearance and reappearance of some clones are common. For instance, no further detection of ST1-V among local clinical CA-MRSA isolates was documented after it was first isolated by Neela et al. (29). Moreover, documentation of ST6-IV, ST5-V, ST508 and ST80-IV showed similar patterns in Malaysia. The recent emergence of ST239-III and ST22-IV in the community added evidence of infiltration of HA-MRSA clones into the community settings.

Another molecular characteristic linked to CA-MRSA is the presence of the PVL gene, which is also true for MRSA isolates in Malaysia. It was reported that 88% to 100% of CA-MRSA isolates in previous studies were positive for PVL gene (21, 28, 29, 36, 54, 55). Nevertheless, there were also reports on PVL negative among CA-MRSA isolates, as listed in Table 1.

## Conclusion

In Malaysia, although HA-MRSA is predominated by ST239-t037-III, its replacement by CA-MRSA clone, ST22-t0-32- IV, is evident. ST30 is the most circulating clone among the community setting. The ease of travelling causes the spread of some clones from their geographical origin to a much wider geographical area. Further to the above, new strains of MRSA have continued to appear and decline for no known reason. Recent findings on the sources of MRSA infection obscure the distinctive characteristics between hospital-acquired and community-acquired strains. Reports on molecular epidemiology of MRSA isolates in Malaysian hospitals have indicated infiltration of the HA-MRSA strain into the community and vice versa, and the PVL gene is increasingly becoming non-exclusive to CA-MRSA isolates. Due to these reasons, comprehensive genotyping for epidemiology and revolutionary study is essential in tracking potential outbreaks, assessing currently adopted precautionary measures and preparing for prophylaxis. In Malaysia, MRSA resistance data is reported consistently through GLASS, NSAR, and other small-scale molecular surveillance by several institutions. Nevertheless, such surveillance efforts are not consistent and not widespread across the country, especially in East Malaysia. A systematic and consistent molecular surveillance of MRSA has proven to lower the dissemination of the pathogen in the European countries (56). Hence, adopting a similar effort could aid in curbing MRSA spread in Malaysia. Currently, researchers worldwide have adopted an in-depth understanding of MRSA epidemiology, innovation and genetic content enabled by whole genome sequencing (WGS). However, such comprehensive use of WGS has never been recorded in Malaysia, as little-to-no local MRSA genome comparison studies have been performed. This could be attributable to the limited access to WGS and the cost of running large-scale WGS beyond the scope of many reference laboratories in Malaysia, especially for genomic data interpretation. With the potential availability of simplified hardware and software packages for genome data generation and analysis, the incorporation of genome analysis in future research and clinical diagnostic use will hopefully be realised in the near future.

## Figures and Tables

**Table 1:** Study on the characteristics and molecular characteristics of HA-MRSA and CA-MRSA isolates from Malaysian hospitals

Reference	Location setting	Study population (sample types)*	Year of study (study period)	No of MRSA isolates	SCC*mec* type**	MLST	*spa* type	Acquisition	PVL gene
Sam et al. (55)	Hospital, multidiscipline ward	Patient exhibiting multi-drug sensitive MRSA isolates (nasopharyngeal aspirate, swab, aspirate, tissue, and blood)	2002–2007	9	IV	ST6	..	CA	−/+
						ST30	..	CA	+
					IVE	ST22	..	CA	−
						ST1178	..	CA	−
						ST1179	..	CA	−
Thong et al. (26)	Hospital, multidiscipline ward	Patient (skin/wound swabs, fluid/secretion, upper respiratory sites, sputum, tissue, bone, and blood)	2003–2004 & 2007	66	III	..	..	..	−
					IV	..	..	..	−/+
					nt	..	..	..	−
Ahmad et al. (28)*****	Reference laboratory, nine hospitals	Outpatient and inpatient (samples from bacteraemia, skin infection, wound infection, abscesses, intravenous line infection, cellulitis)	2006–2008 (20 months)	628	III	..	..	..	..
					IV	ST30	..	HA/CA	+
						ST22	..	HA	+
						ST101	..	HA	−
						ST1285	..	HA	−
						ST1286	..	HA	−
						ST1287	..	HA	−
						ST1288	..	HA	−
						ST45	..	HA	−
						ST188	..	HA	−
						ST1284	..	HA	−
						ST80	..	CA	−
Ghaznavi-Rad et al. (30)	Hospital, multidiscipline ward	Inpatient (pus, cellulitis, abscess, respiratory specimen, blood, medical devices cerebrospinal fluid, conjunctiva, body fluids, urine, and bone marrow)	2007–2008 (12 months)	389	III	ST239	t037	..	−/+
							t421	..	−
							t4150	..	−
							t2475	..	−
							t4213	..	−
					IIIa	ST239	t037	..	−/+
							t421	..	−
							t932	..	−
							t138	..	−
						ST1283	t037	..	−
							t138	..	−
					IVh	ST22	t032	..	−
							t3213	..	−
							t4184	..	−
					V	ST7	t091	..	−
						ST1	t127	..	+
						ST188	t189	..	+
Neela et al. (29)	Hospital, multidiscipline ward	Inpatient (blood, pus, urine, and tracheal aspirates)	2006–2007 (6 months)	36	III	ST239	t037	HA	..
						nt	t074	HA	..
							t3103	HA	..
					IIIa	ST239	t037	HA	..
						nt	t0421	HA	..
					V	ST1	t127	CA	+
Mustafa et al. (57)	Hospital, multidiscipline ward	Inpatient (skin and soft tissue infections, ear-nose-throat infections, respiratory tract infections, blood stream infections, and other body fluids)	2010 (6 months)	28	III	..	..	..	..
					IV	..	..	..	..
Lim et al. (33)	Hospital, multidiscipline ward	Inpatient and healthcare worker (nasal swabs, tissue, wound swabs, urine, pus, body fluids, sputum, nasopharyngeal secretion, catheter tip, bone, blood, and chest tube “drainage”)	2003–2004, 2007–2008	154	..	ST239	t037	..	..
							t421	..	..
							t6405	..	..
							t860	..	..
							t2029	..	..
							t4150	..	..
							t4152	..	..
						ST20	t1544	..	..
						ST5	t002	..	..
						ST6	t304	..	..
						ST22	t032	..	..
							t4184	..	..
							t1378	..	..
						ST80	t037	..	..
						ST541	t363	..	..
						ST573	t458	..	..
Lim et al. (25)	Hospital, multidiscipline ward	Inpatient (swab samples, blood, pus, tissue, urine, sputum, and unknown sites)	2008–2010	35	III	ST239	t037	HA	−
					IV	ST240	t038	HA	−
					V	ST772	t657	HA	−
Rashid et al. (54)	Hospital, multidiscipline ward	Inpatient (pus and wound swab)	2009 (12 months)	5	IV	..	..	CA	+
					nt	..	..	CA	+
Lim et al. (22)	Hospital, multidiscipline ward	Inpatient and healthcare worker (nasal swab, tissue, wound swab, urine, pus, body fluid, sputum, nasopharyngeal secretion, catheter tip, bone, blood, and chest tube “drainage”)	2003 & 2008	162	III	ST239	..	HA/CA	−/+
					IVa	ST22	..	HA/CA	..
					V	ST772	..	CA	−
					IV	ST6	..	HA/CA	−/+
						ST1178	..	HA/CA	..
Muttaqillah et al. (31)****	Hospital, multidiscipline ward	Inpatients and outpatient (swab, blood, pus, tracheal aspirate, nasopharyngeal aspirate, bronchoalveolar lavage, bone, cerebrovascular fluid, and tissue) tissues, sputum, cerebrospinal fluid, and urine)	2009 (12 months)	236	II	ST239	..	..	..
					III	ST239	..	..	..
					IV	ST30	..	..	..
						ST1178	..	..	..
					V	ST772	..	..	+
Noordin et al. (58)	Hospital, multidiscipline ward	Inpatient (wound swab, tracheal aspirate, blood, tissues, sputum, cerebrospinal fluid, and urine)	2009	318	III	..	..	..	−/+
					IV	..	..	..	−/+
					V	..	..	..	−/+
					II	..	..	..	..
					nt	..	..	..	..
					Novel	..	..	..	..
Ho et al. (21)*****	Two hospitals and one private laboratory	Patient (samples from sepsis, skin infections, infected surgical wounds, infected implants, eye infections, bone infections, respiratory tract infections, and unknown condition)	2011–2012	175	III	..	t037	HA	−
						..	t234	HA	−
					IV	..	t304	HA/CA	−/+
						..	t690	HA/CA	−/+
						..	t032	HA	−/+
					V	..	t657	HA/CA	−/+
						..	t345	HA/CA	−/+
					II	..	t2460	HA	−
Sit et al. (10)	Hospital, multidiscipline ward	Outpatient and inpatient (tissues, blood, pus, slough and abscess, cerebrospinal fluid, bone, pericardial fluid, bullae fluid, and synovial fluid)	2011–2012 (24 months)	91	III	ST239	..	HA/CA	−
					IV	ST22	..	HA/CA	−/+
						ST6	..	HA/CA	−/+
						ST1	..	HA/CA	−/+
						ST1137	..	HA/CA	−/+
					V	ST772	..	CA	−/+
						ST5	..	CA	−/+
					II	ST239	..	HA/CA	−
					nt	ST239	..	HA/CA	−
						ST508	..	HA/CA	−
Sit et al. (38)	Hospital, multidiscipline ward	Inpatient (blood)	2013 (12 months)	67	III	ST239	..	HA/CA	−
					I	ST152	..	HA	−
					IV	ST6	..	HA/CA	−
						ST22	..	HA/CA	−
						ST30	..	HA	−
						ST1179	..	HA	−
					V	ST1	..	HA	−
						ST45	..	HA	−
						ST772	..	CA/HA	−
						ST951	..	HA	−
					nt	ST5	..	HA	−
Amit et al. (36)***	Women and children hospital	Paediatrics inpatient (abscess)	2015–2017 (18 months)	37	IVa IVc V II nt	..	t019 t122 t186 t975	CA	+/−
Niek et al. (23)	Hospital, multidiscipline ward	Inpatient (blood, cerebrospinal fluid, subcutaneous hip fluid, bone, and pleural effusion)	2014–2015 (24 months)	99	III	ST239	..	HA/CA	−
					IVa	ST769	..	HA	+
						ST188	..	HA	−
						ST6	..	HA	−/+
						ST45	..	HA	−
						ST22	..	HA	+
						ST88	..	HA	+
					IVc	ST8	..	HA/CA	−
						ST5	..	HA	−/+
						ST45	..	HA	−
					IV (novel subtype)	ST22	..	HA/CA	−
					V	ST772	..	CA	+
						ST3547	..	HA	−
						ST1	..	HA	−
Nik Badrul Alam et al. (59)	Hospital, multidiscipline ward	Patient (blood, pus, endotracheal tube, pleural fluid, sputum, tissue, wound swab, tracheal aspirate, and, slough tissue)	2016–2017 (24 months)	47	..	ST22	..	..	..
						ST239	..	..	..
						ST4649	..	..	..

MRSA = methicillin-resistant *Staphylococcus aureus*; SCC*mec* = staphylococcal cassette chromosome *mec*; MLST = multilocus sequence type; PVL = Panton-Valentine leucocidin; HA = hospital-acquired; CA = community-acquired; = not reported; nt = nontypable; − = negative; + = positive;

* = multisensitive MRSA can be defined as MRSA isolate that is resistant to cefoxitin and sensitive to antibiotics other than beta-lactam antibiotics;

** = the SCC*mec* types were arranged based on the most to the lowest types reported except for the study by Muttaqillah et al. (31) due to their types of sampling strategy;

*** = no correlation between typing, MRSA acquisition and the presence of PVL gene;

**** = PVL gene results were reported based on previous paper;

***** = infection type was reported instead of sample type
